# A Comparison of Spatial and Movement Patterns between Sympatric Predators: Bull Sharks (*Carcharhinus leucas*) and Atlantic Tarpon (*Megalops atlanticus*)

**DOI:** 10.1371/journal.pone.0045958

**Published:** 2012-09-26

**Authors:** Neil Hammerschlag, Jiangang Luo, Duncan J. Irschick, Jerald S. Ault

**Affiliations:** 1 Rosenstiel School of Marine and Atmospheric Science, University of Miami, Miami, Florida, United States of America; 2 Leonard and Jayne Abess Center for Ecosystem Science and Policy, University of Miami, Coral Gables, Florida, United States of America; 3 RJ Dunlap Marine Conservation Program, University of Miami, Miami, Florida, United States of America; 4 Biology Department, University of Massachusetts, Amherst, Massachusetts, United States of America; Macquarie University, Australia

## Abstract

**Background:**

Predators can impact ecosystems through trophic cascades such that differential patterns in habitat use can lead to spatiotemporal variation in top down forcing on community dynamics. Thus, improved understanding of predator movements is important for evaluating the potential ecosystem effects of their declines.

**Methodology/Principal Findings:**

We satellite-tagged an apex predator (bull sharks, *Carcharhinus leucas*) and a sympatric mesopredator (Atlantic tarpon, *Megalops atlanticus*) in southern Florida waters to describe their habitat use, abundance and movement patterns. We asked four questions: (1) How do the seasonal abundance patterns of bull sharks and tarpon compare? (2) How do the movement patterns of bull sharks and tarpon compare, and what proportion of time do their respective primary ranges overlap? (3) Do tarpon movement patterns (e.g., straight versus convoluted paths) and/or their rates of movement (ROM) differ in areas of low versus high bull shark abundance? and (4) Can any general conclusions be reached concerning whether tarpon may mitigate risk of predation by sharks when they are in areas of high bull shark abundance?

**Conclusions/Significance:**

Despite similarities in diet, bull sharks and tarpon showed little overlap in habitat use. Bull shark abundance was high year-round, but peaked in winter; while tarpon abundance and fishery catches were highest in late spring. However, presence of the largest sharks (>230 cm) coincided with peak tarpon abundance. When moving over deep open waters (areas of high shark abundance and high food availability) tarpon maintained relatively high ROM in directed lines until reaching shallow structurally-complex areas. At such locations, tarpon exhibited slow tortuous movements over relatively long time periods indicative of foraging. Tarpon periodically concentrated up rivers, where tracked bull sharks were absent. We propose that tarpon trade-off energetic costs of both food assimilation and osmoregulation to reduce predation risk by bull sharks.

## Introduction

Because movement promotes energy flow across habitat boundaries [1], [2], ecological and evolutionary processes are inherently linked to movement, including ecosystem function and biodiversity [3]. Predicting organismal movement is central to establishing effective management and conservation strategies, such as restoring degraded habitats, reducing exploitation rates, preventing spread of invasive species, and protecting wildlife (i.e. “movement ecology” [4]). A key aspect of movement ecology is interactions among species, especially predators and prey. Dynamics between predators and prey are often complex when considered across relevant spatial and temporal scales [4], [5]. However, growing evidence reveals that predators can regulate ecosystem structure and function via trophic cascades arising through both consumption and predator-induced modifications in prey behavior [6]. Therefore, studies of predator movement patterns are becoming increasingly important for predicting the ecosystem consequences of their declines, especially for marine species that are experiencing significant population declines due to overfishing [7]–[9]. Consequently, further studies of marine predator movements and habitat use are needed to identify and prioritize areas for protection (e.g. feeding and natal grounds) as well as generate sufficient data for modeling how changes in their habitat use can affect sustainability and potentially alter community dynamics [10]–[12].

**Figure 1:**
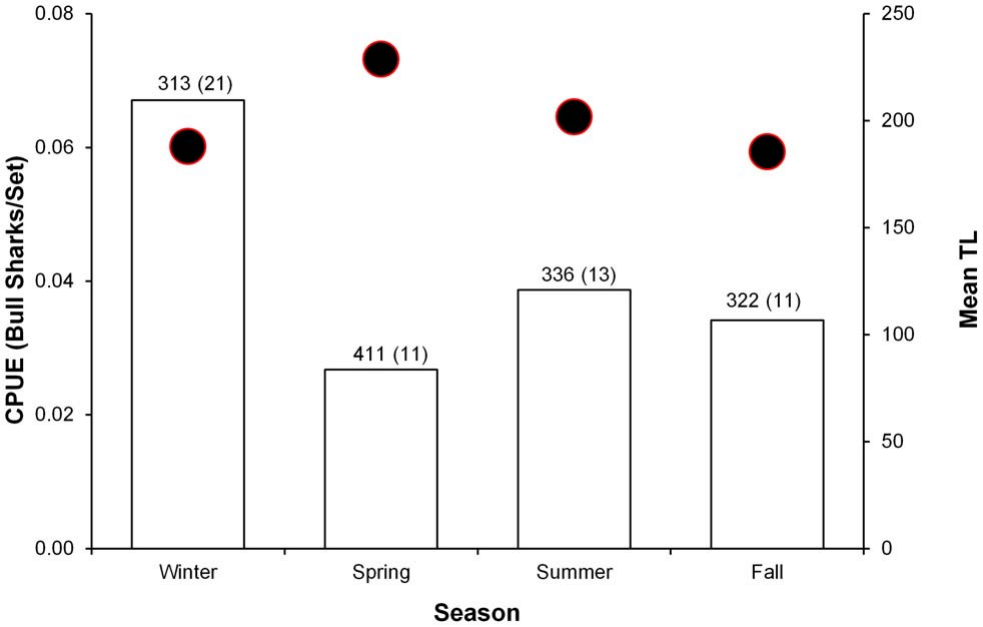
Bull shark catch rates and average size in the catch by season. Data are based on 55 bull sharks captured between October 2009 and May 2012 in the Florida Keys and Florida Bay. Catch per unit effort (CPUE) of drumlines (for all years combined, averaged by season) was used to determine if there were monthly changes in occurrence and size (TL). CPUE was expressed as the number of bull sharks caught per set (bars) and average size of bull sharks caught per set (black dots) within each season (Winter: Dec, Jan, Feb; Spring; Mar, Apr, May; Summer; Jun, Jul, Aug; Fall: Sep, Oct, Nov). Numbers above bars indicate total drumline sets deployed per season (effort) with total bull shark catch per season indicated in parenthesis.

Bull sharks (*Carcharhinus leucas* Müller & Henle, 1839) are apex predators in tropical and subtropical seas [13]–[15]. In the western Atlantic, the species grows to a relatively large size [>340 cm, >230 kg; 34] and occurs from northeastern United States to Brazil. Within this geographic range, bull sharks are common to coastal, estuarine, lagoon and fresh waters, especially certain large lakes and rivers [16], [17]. Bull sharks are unique among elasmobranchs for their ability to inhabit brackish or freshwater systems for relatively prolonged periods due to unique physiological adaptations that permit osmoregulation in low salinity environments [17]–[19]. Studies from Florida and the Gulf of Mexico have found that young of the year and juvenile bull sharks regularly occupy inshore rivers as nursery habitats [20]–[21], but transition out of these areas once they reach about 160–180 cm TL [16]. Gravid females likely return to pup [16]. Only two papers [15], [22] have reported on movement patterns of large (>150 cm TL) bull sharks using archival satellite tags, the latter being the first to describe movements of adult bull sharks in the Gulf of Mexico and southeastern United States. These studies found that adult bull sharks exhibit high site fidelity and primarily utilize shallow coastal zones [16], [22].

Atlantic tarpon (*Megalops atlanticus* Valenciennes, 1847) are highly mobile mesopredators and very popular sportfish [23], [24]. Satellite tagging of Atlantic tarpon in the southeastern United States, Gulf of Mexico and Florida Keys has revealed that, similar to bull sharks, tarpon also tend to utilize inshore coastal, estuarine and freshwater areas where they co-occur [25]–[28]. Bull sharks are commonly observed preying upon tarpon at popular fishing locations in the Florida Keys, southern Florida and Gulf of Mexico during recreational catch and release angling [26]. Examination of bull shark stomachs from the aforementioned region has shown that in addition to tarpon, sharks feed on mullet *Mugilcephalus*, menhaden *Brevoortiapatronis* and ladyfish *Elopssaurus*, all favored food items of the Atlantic tarpon [28]. Given similarities in spatial and trophic niches, tarpon may be susceptible to bull shark predation while foraging.

Here we conducted a joint tagging study of bull sharks and Atlantic tarpon in southern Florida to describe their spatial distribution, habitat use and movement patterns relative to one another. Our first goal was to describe seasonal abundance and general movement patterns of bull sharks and tarpon. Our second goal was to identify core areas of bull shark activity and then examine the movement patterns and swimming behaviors (speed, tortuoisty) of tarpon relative to these core areas of bull shark habitat use. We used these data to address four general questions. First, how do the seasonal abundance patterns of bull sharks and tarpon compare? Second, how do the movement patterns of bull sharks and tarpon compare, and what proportion of time do their primary ranges overlap? Third, do tarpon movement patterns (e.g., straight versus convoluted paths) and/or their rate of movement (ROM) differ in areas of low versus high bull shark abundance? Finally, given the potential for predator-prey interactions, can any general conclusions be reached concerning whether tarpon may mitigate risk of predation by sharks when they are in areas of high bull shark abundance?

**Table 1:** Summary data for 16 bull sharks tracked with SPOT5 tags. TL = Total Length.

Shark ID	TL (cm)	Sex	Tagging Location		Date Tagged	Last Detection	Days at Large
33919	176	F	25.00644	−80.99969	11/7/2010	5/21/2011	194
33937	221	F	24.69740	−80.85227	6/5/2010	10/8/2010	123
33991	210	F	25.00644	−80.99969	3/26/2010	6/20/2010	84
34208	173	M	25.00644	−80.99969	11/7/2010	1/14/2011	67
55492	189	F	26.36898	−81.97914	10/29/2010	1/17/2011	78
55493	216	M	24.69740	−80.85227	8/19/2010	10/21/2010	62
55496	200	M	25.00644	−80.99969	11/6/2010	2/13/2011	97
60695	234	M	25.00644	−80.99969	8/20/2010	9/7/2010	17
60696	170	M	26.36898	−81.97914	10/29/2010	6/11/2011	222
60697	154	M	26.36898	−81.97914	8/10/2010	4/6/2011	236
60698	176	F	25.00644	−80.99969	11/6/2010	1/25/2011	79
60699	195	F	25.00644	−80.99969	10/7/2010	6/11/2011	244
68479	160	M	25.00644	−80.99969	1/28/2011	6/10/2011	132
68483	194	F	25.00644	−80.99969	12/4/2010	2/12/2011	68
105596	245	F	24.81300	−80.90960	2/27/2011	5/9/2011	72
68467	245	M	26.86000	−79.03833	2/19/2011	5/20/2011	91

**Table 2:** Total positions received and corresponding accuracy for each tagged animal.

	Location Class														
	3		2		1		0		A		B		Z		Total
**Shark ID**															
33919	7		8		16		30		13		62		1		137
33937	0		1		0		1		1		28		0		31
33991	3		6		4		4		5		45		1		68
34208	0		1		1		2		1		13		0		18
55492	0		0		0		0		0		0		0		0
55493	0		0		0		0		1		0		1		2
55496	2		3		5		14		6		40		0		70
60695	0		0		0		0		2		1		0		3
60696	1		2		2		5		1		36		2		49
60697	2		0		0		2		4		24		0		32
60698	5		6		11		19		12		71		0		124
60699	13		12		18		25		10		100		0		178
68479	1		0		1		0		1		4		0		7
68483	1		1		2		2		10		60		3		79
105596	7		1		1		2		0		10		0		21
68467	0		2		1		3		1		7		0		14
**Tarpon ID**															
T-176	93		13		6		0		5		42		0		159
T-177	12		7		1		0		4		21		0		45
T-178	13		11		10		1		8		27		0		70
T-179	114		48		11		3		55		203		0		434
T-180	20		5		1		0		1		8		0		35
T-181	1		0		0		0		2		81		0		84
T-182	10		6		3		0		3		6		0		28
T-184	104		54		43		6		22		238		0		467
T-186	17		10		6		2		18		124		0		177
T-187	16		5		1		6		7		96		0		131
T-188	7		7		2		0		5		17		0		38
T-196	37		17		12		1		15		217		0		299

Accuracies are indicated by a location class (LC), ranging in accuracy with the following radius of error: LC 3<250 m, 250 m < LC 2<500 m, 500 m < LC 1<1500 m; median error for LC 0, A and B ranges from 1 to 3 km. Positions with LC Z were excluded from data analyses (See text).

## Methods

### Bull Sharks

Between October 2009 and May 2012, standardized surveys were conducted to capture and tag sharks as part of an ongoing shark abundance and movement study in the Florida Keys (Biscayne Bay, Key Largo, Islamorada, Dry Tortugas) and southeastern Gulf of Mexico (Florida Bay, Everglades National Park, Fort Myers). Sharks were captured using baited circle-hook drumlines as described by Hammerschlag et al [29]. Briefly, sets of 5 drumlines were deployed and left to soak for 1.0 hour before being checked for shark presence. Upon capture, shark sex was recorded, total length (TL) in cm was measured and thereafter, sharks were marked with an identification tag and then released back into the water. Catch per unit effort (CPUE) of drumlines (for all years combined, averaged by season) was used to determine if there were seasonal changes in occurrence and size (TL). CPUE was expressed as the number of bull sharks caught per set and average size of bull sharks caught per set within each season (Winter: Dec, Jan, Feb; Spring; Mar, Apr, May; Summer; Jun, Jul, Aug; Fall: Sep, Oct, Nov).

If a large bull shark (>150 cm TL) was captured during a survey, a satellite telemetry tag was affixed to the sharks’ first dorsal fin. We used Smart Position and Temperature Transmitting (SPOT) tags (SPOT5, Wildlife Computers; www.wildlifecomputers.com) because they provided relatively detailed horizontal movements that could be analyzed at a much higher resolution than light-based position data derived from pop-up archival satellite tags [30]. SPOT tags were coated with Propspeed, a non-toxic, nonmetallic anti-fouling agent, to minimize biofouling [31], [32]. Transmitters were attached using titanium bolts, neoprene and steel washers, and high carbon steel nuts to prevent any metallic corrosion from contacting the fin as well as to ensure that the steel nuts corroded, resulting in eventual tag detachment [33].

**Figure 2:**
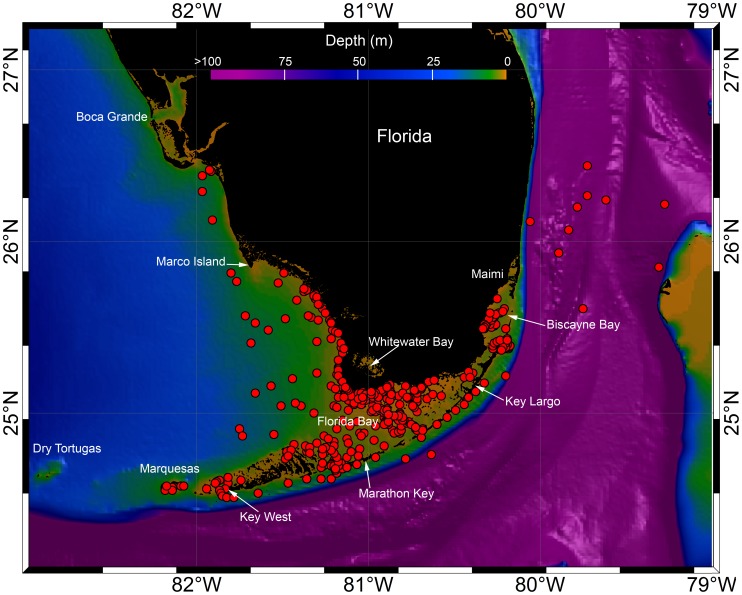
Track positions of 16 bull sharks (red dots) on bathymetry of the study area. All geolocations represent 12 hour interval positions (midnight and noon) filtered using piecewise Bézier interpolation. Depths are scaled from 0 to 100 m as indicated by the color bar.

**Figure 3:**
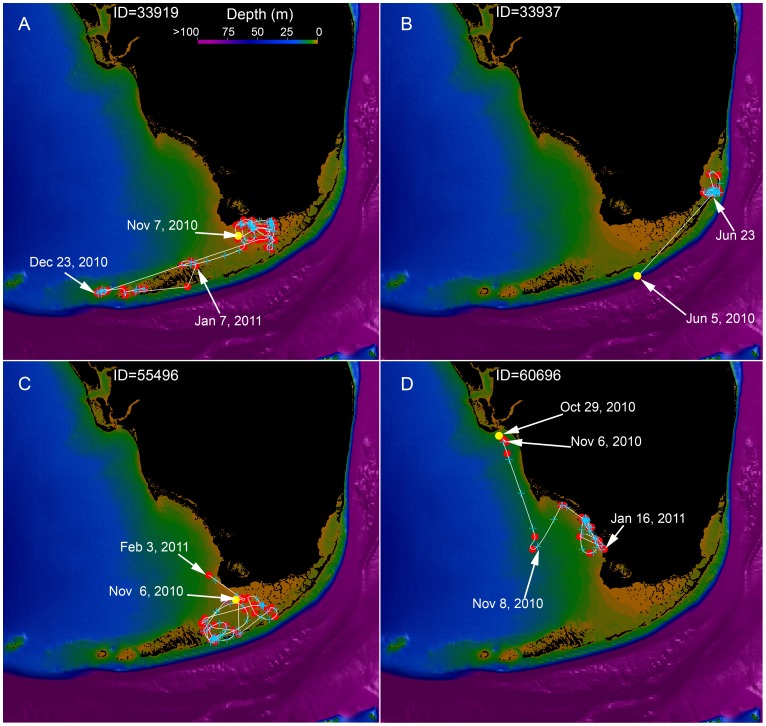
Representative example of four different bull shark tracks overlain on regional bathymetry. Red dots indicate the Argos locations, the white line is the 1-minute Bezier interpolated track, and the blue plus symbols are the 12-hour positions. The yellow dot is the tagging location. The depth is scaled from 0 to 100 m as indicated by the color bar. Major geographic locations are labeled in Fig. 1.

### Tarpon

Data on seasonal abundance patterns for tarpon were obtained from two sources: (1) the Cooperative Tagging Center (CTC) based at NOAA’s National Marine Fisheries Service, Southeast Fisheries Science Center, Miami, Florida [34], [35]; and, (2) creel surveys of professional fishing guides in Everglades National Park acquired from the National Park Service, Homestead, Florida [36]. Release locations of conventionally tagged tarpon from 1962 to 2004 derived from the CTC database were plotted with ArcGIS by season (Winter: Dec, Jan, Feb; Spring; Mar, Apr, May; Summer; Jun, Jul, Aug; Fall: Sep, Oct, Nov) and by size (weight in kg). In addition, numbers of tarpon caught by recreational fishers in ENP by month, averaged for the period 1980–2006, were extracted from the creel survey database.

Between March 2011 and June 2011, tarpon were captured for satellite tagging using standard hook-and-line gears on chartered recreational fishing boats in the southern Florida Keys (Islamorada, Bahia Honda), Biscayne National Park (Broad Key), Everglades National Park (Whitewater Bay), Boca Grande and southeastern Gulf of Mexico. Upon capture, tarpon fork length (FL) and girth (G) were measured in cm and weight in kg was computed with the algorithm of [37]; thereafter, a SPOT tag was attached to the tarpon’s body via a 40 cm long stainless steel wire tether to a titanium anchor dart. The anchor dart was inserted into the flank of the tarpon about 15–20 cm anterior to the dorsal fin and roughly 5–10 cm above the lateral line.

**Figure 4:**
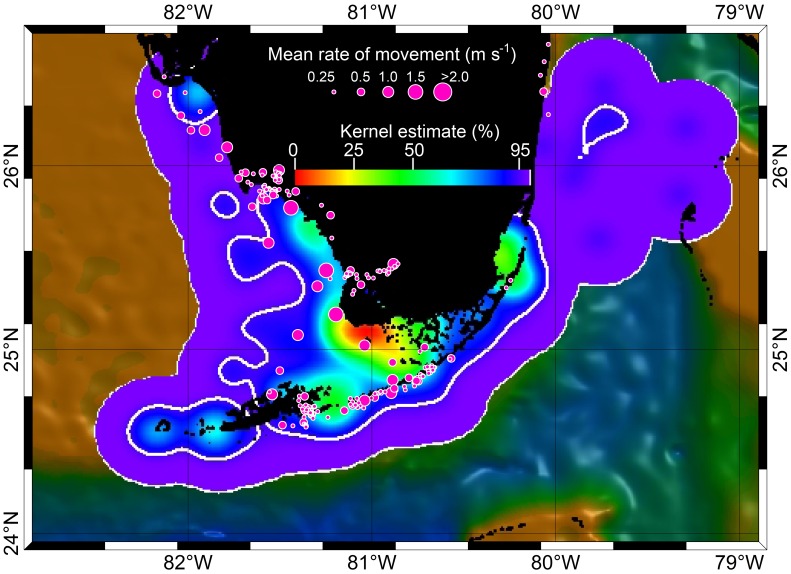
Predator-prey interactions between bull sharks and Atlantic tarpon in the southern Florida ecosystem. Colored contours indicate summarized accumulative kernel density values (from high density to low density) for 16 bull sharks and sizes of circles represent tarpon locations and rate of movement (m s^−1^). A tarpon (T182) released on 23 May, 2011, was likely attacked (red triangle) by a shark on 28 May in the core area of shark use (see text).

### Movement Data Analysis

The geographic location of satellite-tagged sharks and tarpon were determined by Doppler-shift calculations made by the Argos Data Collection and Location Service, www.argos-system.org) whenever a passing satellite received signals from the tag at the surface. To improve location accuracy, we processed all Doppler derived data using Kalman filtering (KF). Argos provides the following radius of error for each KF-derived location class (LC): LC 3<250 m, 250 m < LC 2<500 m, 500 m < LC 1<1500 m; Argos states that the median error for LC 0, A and B ranges from 1 to 3 km [38]. Class Z indicates that the location process failed and estimates of position are highly inaccurate. All transmitted locations were filtered to remove positions with LC Z, those on land, and those exceeding a speed of 2 m/s (following Weng et al [39]). Argos-derived locations were plotted using ESRI ArcGIS 9.3.

We performed utilization distribution analyses on position data using fixed kernel density metrics. Kernel density estimates quantify the core regions of occupancy within an animal’s home range or activity space [20], [40]. Kernel density values are cumulated from the highest to lowest density areas to create kernel density contours. Thus, the 25% contours represent areas of the top highest observed densities, while the 95% contours represent up to 95% density areas. These metrics were calculated according to the equations provided by Worton [41] and plotted using Interactive Data Languages (IDL, www.ittvis.com) software. Following Domeier and Nasby-Lucas [40] and Weng et al [42], kernel density estimates were calculated for all sharks grouped as species-specific habitat utilization instead of the individual’s home range.

**Figure 5:**
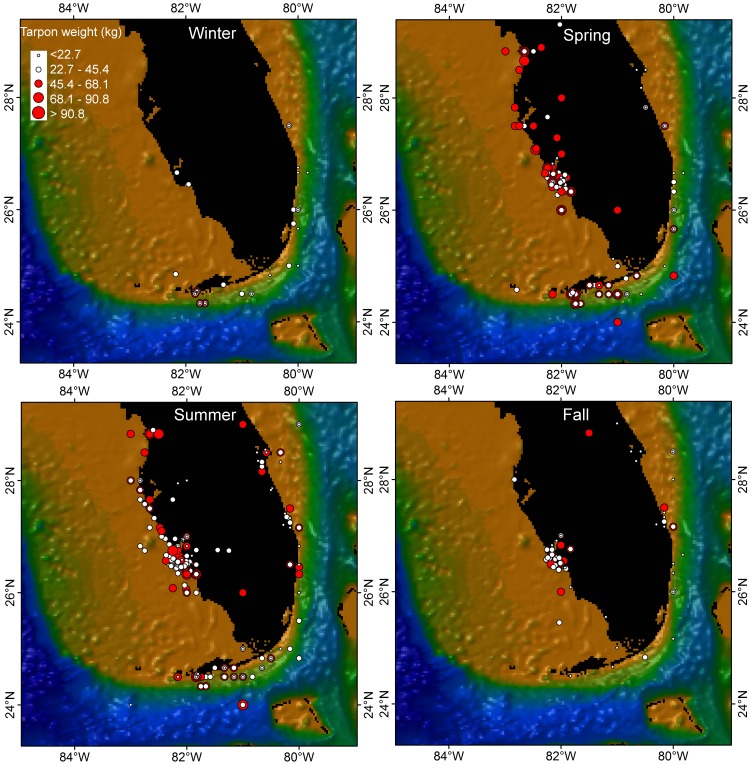
Release locations of conventionally tagged Atlantic tarpon in South Florida from 1962 to 2004 in Winter (a), Spring (b), Summer (c), and Fall (d). The sizes of the tagged tarpon were grouped into 5 weight classes (<22.7, 22.7–45.4, 45.4–68.1, 68.1–90.8, >90.8 kg) are indicated by the size of dots as shown in the legend. Tarpon <90.8 kg were indicated by white dots, and >90.8 kg were indicated by red dots. Winter: Dec, Jan, Feb; Spring; Mar, Apr, May; Summer; Jun, Jul, Aug; Fall: Sep, Oct, Nov).

**Figure 6:**
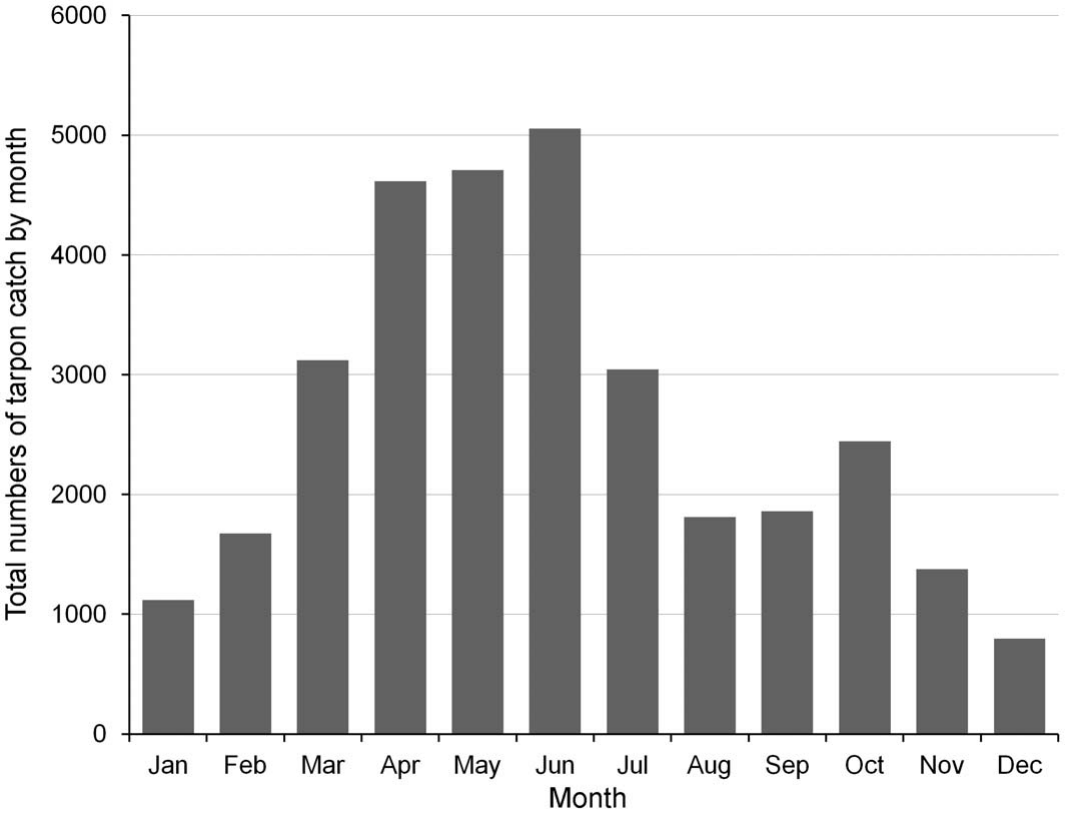
Average total numbers of tarpon caught by month by recreational anglers in Everglades National Park from 1980 to 2006. Data based on National Park Service Creel Surveys of professional fishing guides in Everglades National Park [36].

Kernel estimates cannot be conducted on SPOT-derived raw data because of the irregular sampling intervals at which data are acquired, gaps in data, and autocorrelations due to successive locations [43]. To account for these biases, filtered tracks were regularized to a frequency of 12 hour intervals (midnight and noon), using piecewise Bézier interpolation methods similar to Tremblay et al [44], but modified with the algorithm by Lars Jenson (http://ljensen.com/bezier/). We employed the modified algorithm to eliminate unnatural loops in the tracks that occur with Bezier method used in [44]. Interpolating track sections with large temporal gaps increases uncertainty (reduces confidence) in data. To explicitly deal with this, we did not interpolate gaps in the data that exceeded three days following the methods of [42].

To describe potential interactions between sharks and tarpon, ROM and tortuosity of tarpon movements were compared relative to bull shark core areas of occupancy (i.e., shark kernel densities) by applying generalized linear models [45]. ROM was calculated as the linear distance traveled in 12 hours. We used the VFractal d [46] as a metric of movement tortuosity. VFractal d values were calculated as a function the turning angle for each pair of consecutive movements described in detail by Nams [46]. ROM and VFractal d were calculated based on the filtered interpolated positions. VFractal d is different, but similar to, fractal d (each point versus each track) which can be estimated by calculating the mean of VFractal d values of all location points for each tarpon movement track [46]. In this study, we used only the VFractal d values, not the fractal d.

**Figure 7:**
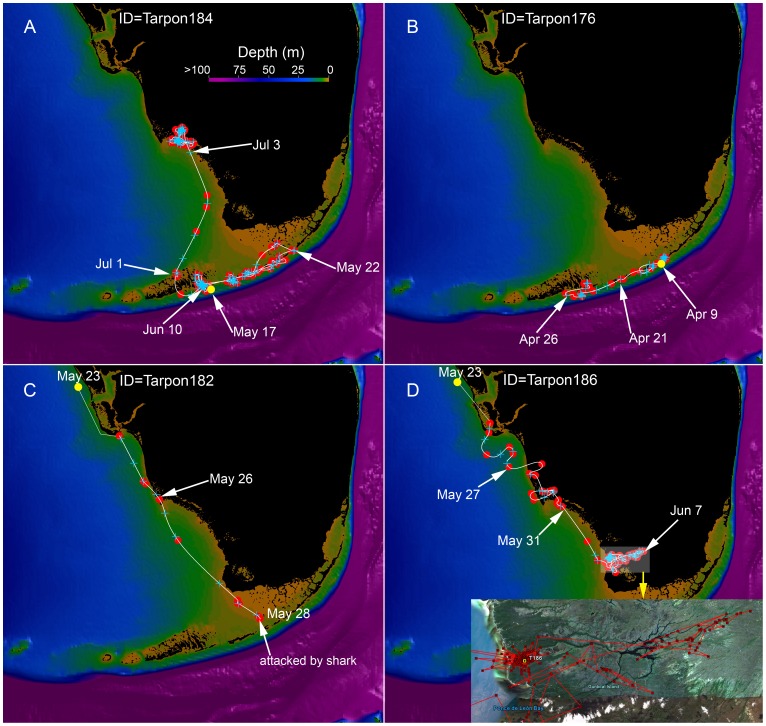
Representative example of four different tarpon tracks overlain on regional bathymetry. The red dots indicate the Argos locations, the white line is the 1-minute Bezier interpolated track, and the blue plus symbols are the 12-hour positions. The yellow dot is the tagging location. The depth is scaled from 0 to 100 m as indicated by the color bar. Major geographic locations are labeled in Fig. 1.

**Figure 8:**
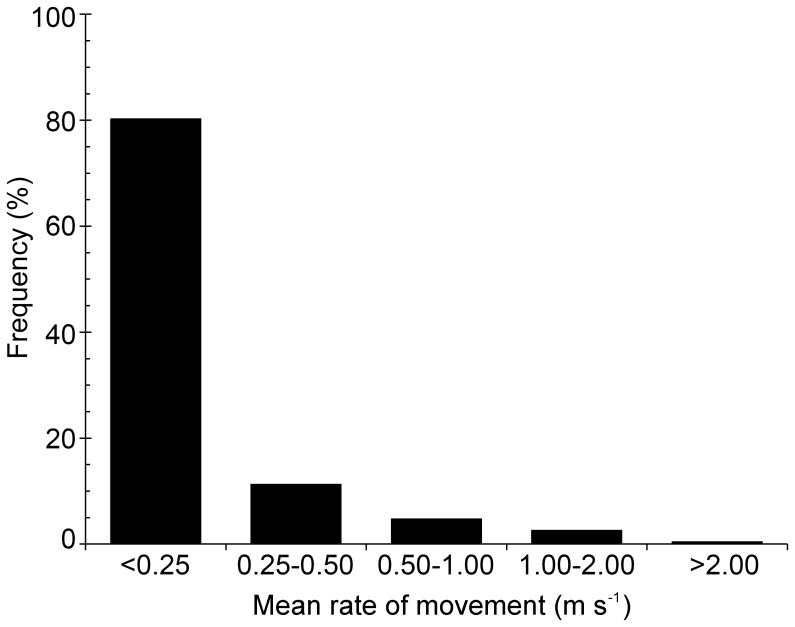
Distribution of tarpon mean rate of movement derived from 12-hour SPOT-tag location data.

**Figure 9:**
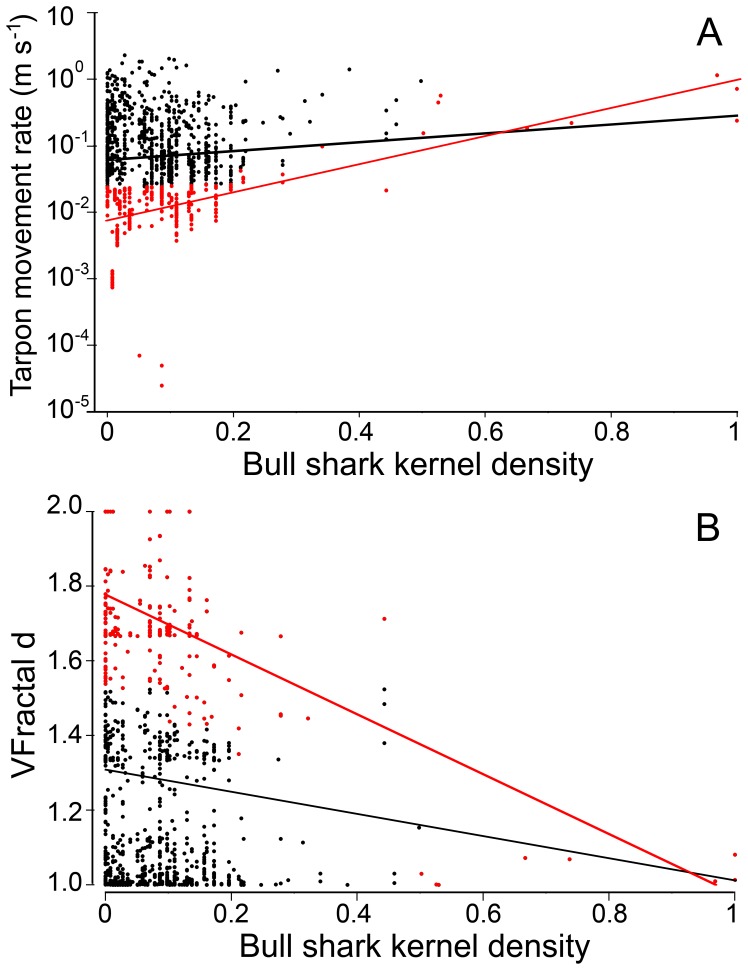
Tarpon movement rate (a) and tarpon track tortuosity (*VFractal d*) (b) as a function of bull shark kernel density. Two analyses were conducted: one with all tarpon data (black and red dots) overlapped with bull shark distribution range; the other with bottom 25% of movement values (red dots in (b)) or top 25% of VFractal d values (red dots in (b)) for each 0.1 bin of bull shark kernel density. The bull shark kernel densities were re-scaled from 0 to 1.0 for low to high density and movement rates were transformed with log10.

**Table 3:** Summary data for 10 Atlantic tarpon tracked with SPOT5 tags.

Tarpon ID	FL (cm)	G (cm)	W (kg)	Latitude	Longitude	Tag Date	Days at Large
T-176	199	99	78.8	24.8469	−80.7508	9/4/2011	20
T-177	155	92	46.5	24.8411	−80.7503	2/4/2011	6
T-178	175	94	59.0	25.2908	−81.0161	03/19/2011	8
T-179	186	100	70.9	25.3524	−80.2595	04/27/2011	60
T-180	172	75	43.4	25.3524	−80.2595	04/28/2011	12
T-181	180	90	58.8	26.7500	−82.1580	06/15/2011	39
T-182	150	78	35.8	26.6322	−82.2366	05/23/2011	5
T-184	172	90	54.3	24.6576	−81.2874	05/17/2011	62
T-186	150	76	34.7	26.6310	−82.2369	05/23/2011	30
T-188	155	78	37.9	25.7580	−80.1293	8/6/2011	6

FL = Fork Length; G = Girth.

## Results

During shark surveys, we deployed 1,382 standardized sets, in which 3,699 individual drumlines were deployed. During these sets, 815 sharks were caught, of which 56 were bull sharks, ranging in size from 142–269 cm TL (average 200 cm TL). Bull shark catch rates and average size by season are plotted in Figure 1. Bull sharks were caught year round, but catch rates were highest in December and January; although the largest sharks (>230 cm TL) were caught between April and July.

Between March 2010 and February 2011, 18 bull sharks were SPOT tagged off southern Florida. Of these, a total of 16 (8 male and 8 female) transmitted valid geolocations that permitted movement tracks to be evaluated. Sharks ranged in size from 154–245 cm TL (average 197.4 cm TL, Table 1). Accuracies of spatial locations ranged from <250 m to <3 km (Table 2). No sharks within the dataset moved into inshore rivers.

Bull sharks exhibited high site fidelity, primarily restricting movements to shallow inshore areas where they were tagged (Fig. 2, 3). Only one shark (194 cm female, # 68483, Table 1) made a relatively long-distance migration. Initially tagged in Everglades National Park (17 miles west of Islamorada), this shark traveled northwest into the Gulf of Mexico over the course of 10 days and after approximately one month, it returned to the Florida Keys. Over the next month, the shark moved northward along the Florida Keys crossing the Straits of Florida to the Bahamas, swimming to the vicinity of Bimini. The shark then traveled southeast, again crossing the Straits of Florida before entering Biscayne Bay when transmissions ceased a month later. The minimum straight line distance of this 68 day trip was approximately 1,200 km.

The fixed kernel results for tagged bull sharks, displayed as volume contours, showed that a core area of 670 km^2^ (25% kernel contour) centered at the northwestern region of Florida Bay (Fig. 4). The 50% kernel contour (2,260 km^2^) indicates the areas of moderate use extended out to most of Florida Bay, Florida Keys and the Biscayne Bay (Fig. 4). The 95% kernel contour (18,042 km^2^) shows the areas of 95% habitat utilization by our tagged bull sharks. We consider areas where bull shark kernel densities exceeded 50% to be “high density” zones, whereas areas where kernel densities were less than 50% were “low-density” zones.

Tarpon were captured year-round by recreational anglers in southern Florida waters; however, strong seasonal differences in catch rates and sizes of animals caught were found (Fig. 5, 6). Large mature fish (>45.4 kg) appear to be virtually absent from the region in winter (early December-late March). The bulk of the migratory front arrives in late spring (mid- to late-April) and departs the area (going northward) by early-summer (late June) (Fig. 5). There is a secondary surge of catch rates in fall as tarpon travel southward through the area during the October to mid-November period (Fig. 5). Other tarpon caught during the year are largely immature fish that tend to use the local rivers and estuaries. Creel data derived from surveys of anglers fishing in Everglades National Park showed the same bi-modal pattern in catches and catch rates (Fig. 6). Tarpon catches were lowest from November through February; highest from April to a peak in June, declining from July through September, and then with a secondary peak again in October (although to a much lesser extent than in early summer).

Tarpon that were satellite-tagged ranged in size from 150–199 cm FL (average 169.4 cm FL, Table 3). Accuracies of spatial locations were similar to those for sharks (Table 2). Of the 10 tarpon tracks, three were on the east coast of Florida, two in the Florida Keys, and five along the west coast of Florida (Fig. 4, 7). The first three tracks (T-176, T-177, T-178) were relatively short due to apparent tag failures. The other three short tracks (T-180, T-182, T-188) were most likely a result of shark attack (Fig. 4, see discussion for more details). Relatively few tarpon tracks, in relation to bull shark tracks, were distributed over open or deep waters (Fig. 4, 7). In contrast, tracks of tarpon, relative to bull sharks, were clustered around shallow Keys and passes. Moreover, tarpon tracks were also concentrated up rivers, where tracked bull sharks were absent (Fig. 4, 7). Tarpon ROM were highest (>1 m/s) where bull shark kernel densities were highest (<50% kernel contour) and ROM were slowest (<0.5 m/s) where shark kernel densities were lowest (>50% kernel contour, Fig. 4). Tagged tarpon spent most of their time (>90%) swimming at relatively low ROM (<0.5 m/s, Fig. 4, 8), coinciding with areas where shark kernel densities were lowest (kernel contours >50%, i.e., “low-density” zones). In contrast, tarpon spent little time (<4%) swimming at high ROM (>1 m/s), coinciding with areas where shark kernel densities were high (kernel contours <50%, i.e., “high-density” zones, Fig. 4, 8). This is statistically supported by the positive correlation from the regression model of tarpon ROM dependent on bull shark kernel density (Fig. 9 a). To inspect the data at different levels, two statistical analyses were conducted: (1) with all tarpon data (black and red dots in Fig. 9 b) overlain on bull shark distribution; and, (2) with bottom 25% of ROM values (red dots) for each 0.1 bin of bull shark kernel density. In these analyses, bull shark kernel densities were rescaled from 0 to 1.0 for low to high density and ROMs were transformed by log10. In both analyses, correlations from the regressions were statistically significant: for all data correlation coefficient (r) was 0.1035 (P<0.005, intercept (b_0_) = −1.239, slope (b_1_) = 0.6869), and for the bottom 25% data r = 0.5806 (P<0.0001, b_0_ = −2.1233, b_1_ = 2.1499).

The tortuosities along tarpon movement tracks were negatively correlated with bull shark kernel density (Fig. 9 b). Similar to the ROM analysis, two additional analyses were conducted for VFractal d data: one with all tarpon data (black and red dots in Fig. 9 b) overlapped with bull shark distribution range; the other with top 25% of VFractal d values (red dots) for each 0.1 bin of bull shark kernel density. In both analyses, these correlations were statistically significant: for all data r = −0.093 (P<0.005, b_0_ = 1.3021, b_1_ = −0.2917); and, for the top 25% data r = −0.5887 (P<0.0001, b_0_ = 1.7762, b_1_ = −0.8009). These results indicated that tarpon generally used low tortuous (i.e., straight-line) movement patterns in shark high-density zones, and used high tortuous movement patterns in shark low density zones.

**Figure 10:**
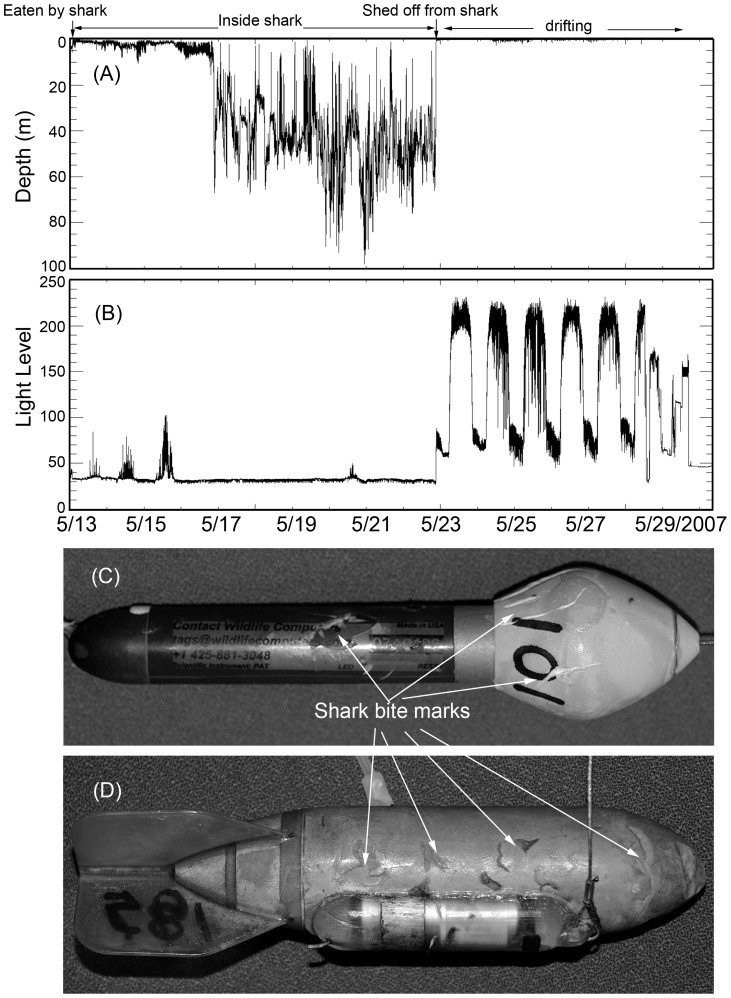
Data recorded by a PAT tag deployed on a mature tarpon between May 13^th^ and 29^th^, 2007, showing: (a) depth; and, (b) light-level recorded every second. The low to absent light-levels shown in panel (a), and the abrupt changes in depth shown in panel (b) indicate the tag was likely ingested by a shark from just after release May 25^th^. Examples of recovered tags from tagged tarpon that had likely fallen prey to sharks: (c) PAT tag; and, (d) SPOT tag, both bear the tell-tale teeth marks (based on spacing and serration) of a shark. Although we cannot identify the species of shark by the bite marks on the tag, it seems plausible that a bull shark was responsible given that the other large shark species are relatively rare in the region, whereas the attack occurred at the location of highest bull shark density in the area.

## Discussion

Our study had several key findings. First, bull sharks were present in the ecosystem year round; but, abundance was generally higher in the winter. In contrast, tarpon catches were highest in early summer with a secondary peak in the early fall. However, presence of the largest bull sharks (>230 cm) coincided with peak tarpon abundance. Second, bull sharks and tarpon generally occupied different aquatic habitats despite similar trophic niches. Bull sharks preferred shallow marine habitats close to the coast of Florida; while tarpon preferred estuarine and riverine regions, with only occasional forays into deeper marine waters where bull shark abundance was greatest. Third, the locomotor behavior and ROMs of tarpon also differed notably between inland riverine habitats and the more open coastal marine habitats. Specifically, tarpon approximately doubled their average ROM in marine coastal regions where bull sharks appeared to concentrate. Finally, tarpon also had straighter and more direct paths in areas of high bull shark patch use and more convoluted paths in areas of low bull shark use. We propose several hypotheses relating to optimal foraging strategies of both tarpon and bull sharks to explain these observed patterns.

At a regional scale, tarpon migration is likely driven principally by water temperatures and prey abundance [25], [26]. Tarpon migrate characteristically with the 26°C isotherm, for example, which passes northward through southern Florida waters during the period of mid-April to late May each year. The timing of large mature tarpon movement into Florida Bay and the Florida Keys is coincident with the spawning event (i.e., specifically the process of building the gonad just before spawning, and ensuring survivorship of the fertilized eggs to larvae via biophysical factors) and feeding (building the soma for survivorship, and preparing the long northward migrations ahead) [25], [26].

The core area of bull shark activity found within northwestern area of Florida Bay is likely driven by the high abundance of teleost prey concentrated there. By conducting shark and fish surveys throughout Florida Bay, Torres et al [47] found that the abundance of seven species of sharks (including bulls) in the northwestern area of Florida Bay was highly correlated with the abundance of 45 teleost species. Given tarpon feeding habits, we would have similarly expected tarpon habitat use to have also been relatively high within the northwestern area of Florida Bay. However, tarpon movements were suggestive of avoiding this area (low residence and high rate of movement in directed lines). In contrast, tarpon exhibited highly tortuous movements over relatively long time periods along the outskirts of Florida Bay as well as in adjacent rivers, which is indicative of foraging, although prey abundance patterns are relatively low in these areas compared to the northwestern area of the Bay.

Productive habitats that contain the greatest food resources are often inherently dangerous for prey, thus creating the need for prey to modify their locomotor behavior and habitat use in response to the threat of predation [48]–[50].The observed movements by tarpon in Florida Bay are suggestive of a food-risk trade-off. For example, studies with lizards and rodents [51] have each shown that they tend to use a bimodal distribution of locomotor speeds, with slower speeds in more protected, safer, habitats and faster speeds in more open, risky, habitats. Desert lizards (*Uma scoparia*) move slowly along convoluted paths underneath vegetation when undisturbed, which likely shields them from both overheating and from predators, but they then move rapidly in direct lines in open areas [52]. Given that prey can elude predators by escaping into a refuge, moving through exposed habitats results in dramatically increased locomotor effort [53]–[55]. This pattern is consistent with the alterations in speed by tarpon in areas of high and low bull shark density observed in this study.

We suggest that another trade-off may be associated with the additional metabolic costs incurred by tarpon that occupy brackish or freshwater zones where bull shark density is low. Generally, the energetic costs of osmoregulation in teleost fish are higher in freshwater than seawater (e.g., Febry and Lutz [56]). The energetic expense occurs because of the need to maintain fluid volume balance by excreting the extra water, while at the same time, trying to conserve internal ionic balance, a biological process which is energetically expensive ([56]; G. Anderson, personal communication). The fact that tarpon spend relatively little time in what would appear to be more optimal coastal marine habitats (from both a food and osmotic perspective), and move so quickly through them, further suggests that these habitats may be risky for them.

It is worth noting that our own anecdotal observations indicate threat of predation mortality to tarpon in areas of high bull shark use. For example, Tarpon T-182 was tagged and released on May 23, 2011, in an area of low bull shark density. The tarpon moved southward through Florida Bay and into a bull shark high density area, at which point it was likely attacked and consumed by a shark on May 28th (Fig. 4, 10). This presumption is based on two factors. First, the depth and light-level data derived from the recovered tag is indicative of being ingested (Fig. 10 a,b). Additionally the recovered tag displayed scratch marks that appear to have been inflicted by a shark based on tooth spacing and serration (Fig. 10 c,d). Although we cannot identify the species of shark by the bite marks on the tag, we believe only tiger (*Galeocerdo cuvier)*, hammerhead (*Sphyrna sp.*) and bull sharks are likely candidates for attacking a large tarpon (and severing the tag’s stainless steel tether). However, it seems plausible that a bull shark was responsible given that the former two species are relatively rare in the region, whereas the attack site represents the location of highest bull shark density in the area.

Critical examination of bull shark diet from the region is limited [28] and although tarpon have been found in bull shark stomach contents, there exists little evidence of bull sharks routinely targeting tarpon as preferred prey. In contrast, bull sharks are commonly observed preying upon tarpon in the region during recreational catch and release angling [26]. Therefore, we hypothesize that a behaviorally mediated indirect interaction (BMII; reviewed by [57]) may be occurring between sharks and tarpon. Specifically, we speculate that higher shark abundance in the northwestern area of Florida Bay is largely driven by relatively high teleost abundance (preferred prey) there [47], which in turn, indirectly causes tarpon to reduce their use of this productive area when foraging to minimize their risk of potential mortality by sharks. A similar BMII has been described in Shark Bay, Western Australia, among tiger sharks, duogongs (*Dugong dugon*), dolphins (*Tursiops aduncus*), turtles (*Chelonia mydas*) and cormorants (*Phalacrocoraxv arius*) [58]. Here, seasonal presence of dugongs (preferred prey of tiger sharks) in shallow waters during summer results in peak tiger shark abundance in these habitats. This, in turn, causes dolphin, turtles and cormorants (species not routinely attacked by sharks) to reduce their use of these productive habitats during summer to minimize risk of potential predation [58]. That said, our hypotheses outlined above require significant investigation by increasing tracking efforts and gathering further ecological data for sharks, tarpon and their potential prey. For example, greater confidence in our hypotheses would be achieved if changes in the spatial and/or temporal movements of sharks corresponded with compensatory adjustments in tarpon swimming behavior and distribution in areas previously occupied by sharks [59]. Because movement patterns in animals are complex and can be influenced by many different variables, our study cannot directly reveal whether the movements of tarpon or bull sharks influence one another *per se*. Tarpon seasonal migrations are likely cued to the changes in water temperature in combination with the movement and distribution of prey [25]. Therefore, the observed tarpon swimming behavior could also be driven by other factors or the combination of them such as environmental preferences (temperature and salinity), feeding needs, and reproductive behaviors [25].

Although use of SPOT tags provided spatial data at higher resolution than archival tags, the major limitation of using Argos-derived data from SPOT tags is the need for animals to surface for long enough to allow successive transmissions for obtaining accurate positions and, therefore, estimating fine scale measurements of speed and fractal values. This is problematic because sharks and tarpon surface irregularly and thus can generate gaps in data acquisition and autocorrelation due to consecutive positions [43]. To overcome this issue, we used filtered tracks that were regularized to a frequency of 12 hour intervals using interpolation. Ideally, it would be better to use higher resolution temporal data (i.e. <12 hrs) if sharks and tarpon transmitted frequently; however, we found that a 12 hr intervals was optimal in this study based on the frequency of transmissions received. Further, given the limitations in estimating tarpon versus bull shark density, results were strongly influenced by several high shark density values; however, these data were not outliers, but the analysis (and its interpretation) would benefit from a larger data set. We are aware that it would have been ideal to analyze potential overlap in kernel densities between tarpon and sharks. However, since tarpon were concentrated up inland rivers, kernel density estimates calculated would have indicated primary activity space over land, therefore negating such a comparison. Additionally, kernel density estimates for bull sharks could have been biased to the site of tagging, and although this cannot be ruled out, we believe it is unlikely since sharks were tagged throughout the middle keys on both the Atlantic and Gulf coasts (where they also transmitted). Further, restricting focus on data derived from Florida Bay, where shallow water depths likely favored transmission, would not impact the general conclusions drawn from this work. Another potential shortcoming of this study worthy of consideration is that tracking period and duration for tarpon was shorter than for sharks, making our discussion on predator-prey interactions somewhat speculative. Also, positional data used varied in accuracy from less than 250 m up to 3 km. However, we believe that this error scale, when compared to the scale of shark and tarpon movements, was sufficient to describe the spatial habitat use patterns observed.

Investigating the movements and fine scale foraging behaviors of marine predators presents several formidable biological and logistical challenges. Future investigations of this kind in marine systems will benefit from employing multiple types of animal-borne instrumentation and sensors (e.g. video, accelerometers, satellite and acoustic telemetry, etc.) to better understand and quantify dynamic interactions among marine predators and between highly mobile fishes and their prey [60]. Given their relatively high site fidelity in shallow near shore waters, both bull sharks and tarpon may be disproportionately vulnerable to coastal fishing and other anthropogenic impacts including reduced water quality, pollution, reductions in their prey, and habitat modifications. Accordingly, increasing studies of these and other marine predator movement patterns are needed to identify and prioritize areas for protection as well as for predicting how anthropogenic-driven changes in their habitat use may impact ecosystem dynamics and vice versa.
